# Evaluation of the Efficacy of Human Dental Pulp Stem Cell Transplantation in Sprague–Dawley Rats with Sensorial Neural Hearing Loss

**DOI:** 10.1055/s-0043-1761190

**Published:** 2023-01-30

**Authors:** Visut Rawiwet, Rattanavijit Vijitruth, Chareonsri Thonabulsombat, Kutkao Vongsavan, Hathaitip Sritanaudomchai

**Affiliations:** 1 Central Animal Facility, Faculty of Science, Mahidol University (MUSC-CAF), Bangkok, Thailand; 2 Department of Biology, Faculty of Science, Mahidol University, Bangkok, Thailand; 3 Department of Anatomy, Faculty of Science, Mahidol University, Bangkok, Thailand; 4 Department of Pediatric Dentistry, Faculty of Dentistry, Mahidol University, Bangkok, Thailand; 5 Department of Oral Biology, Faculty of Dentistry, Mahidol University, Bangkok, Thailand

**Keywords:** dental pulp stem cells, neuronal cell differentiation, sensorineural HL, spiral ganglion neurons

## Abstract

**Objectives** The purpose of the present study was to evaluate the efficacy of spiral ganglion neuron (SGN) regeneration after dental pulp stem cell (DPSC) transplantation in a rat sensorineural hearing loss (HL) model.

**Materials and Methods** Sham or experimental HL was induced in adult Sprague–Dawley rats by cochlear round window surgery. An HL rat model was established with a single 10 mM ouabain intratympanic injection. After 7 days, the rats received DPSCs, stem cells from human exfoliated deciduous teeth (SHED), or culture medium in the sutural area to establish four groups: sham, HL-DPSC, HL-SHED, and HL-medium. Histological analyses were performed at 4, 7, and 10 weeks after transplantation, and the number of SGNs, specific SGN protein expression, and the function of SGNs were evaluated.

**Statistical Analysis** Data were statistically by MS Excel and SPSS v.15.0. Intergroup level of significance was determined via a one-way analysis of variance and Duncan's multiple range test with 95% confidence intervals.

**Results** New SGN formation was observed in the HL-DPSC and HL-SHED rat groups. The number of SGNs was significantly higher in the HL-DPSC and HL-SHED groups than in the HL-medium group over 4 to 10-week survival period. HL-DPSC rats exhibited higher SGN density compared with that in HL-SHED group, which was statistically significant at week 10. The regenerated SGNs expressed cochlear wiring regulator GATA-binding-protein 3. Moreover, the SGNs from the HL-DPSC group also exhibited a higher expression of synaptic vesicle protein and regulated action potential-dependent neurotransmitter release compared with SGNs from the HL-SHED group.

**Conclusions** Our findings suggest that DPSCs and SHED repair and regenerate SGNs in rat HL model. Dental pulp stem cells represent a promising treatment strategy for restoring damage to the sensory circuits associated with deafness.

## Introduction

The World Health Organization (WHO, 2021: http://www.who.int/news-room/fact-sheets/detail/deafness-and-hearing-loss ) reported that, globally, more than 5% population requires treatment for disabling hearing loss (HL) (432 million adults and 34 million children). Nearly 80% of people with disabling HL live in low- and middle-income countries, particularly in sub-Saharan Africa and South and Southeast Asia. 1 Sensorineural HL (SNHL) is a major problem and is typically brought about by damage to the spiral ganglion neurons (SGN) or the hair cells in the cochlea. 2 Many factors are etiologically associated with the degeneration processes of these structures, including aging, heredity, and environmental stimuli (e.g., loud noises or ototoxic drugs). SGNs are strung along the bony core of the cochlea. The dendrites of SGNs contact the base of hair cells synaptically and send fibers (axons) into the central nervous system. These cells cannot regenerate after destruction, which results in their permanent loss. Several types of stem cells have been used to treat SNHL, including embryonic stem cells 3 4 5 6 7 and bone marrow-derived mesenchymal stem cells. 8

Dental pulp stem cells (DPSCs) and stem cells from human exfoliated deciduous teeth (SHED) are promising alternative cell sources for the treatment of neurodegenerative diseases. DPSCs and SHED are ectomesenchymal stem cells and are capable of growing and developing into progenitor cells in the neural crest. 9 10 11 DPSCs from both young and adult teeth are able to differentiate into SGN-like cells after exposure to inner ear neurotrophins, such as brain-derived neurotrophic factor, neurotrophin-3 (NT-3), and glial cell-derived neurotrophic factor. 12 Moreover, co-cultures of DPSCs or SHED-derived neural progenitor cells (NPCs) with auditory brainstem slices promote SGN differentiation. 13 Neuronal cell differentiation is accompanied by high expression of the specific SGN markers tyrosine receptor kinase B, GATA-binding protein 3 (GATA3), and synaptic vesicle protein (SV2A), and oscillations of intracellular calcium occur during the process. However, the ability of DPSCs and SHED to regenerate SGNs in the cochlea of animals with SNHL has not been reported.

In this study, we evaluated the ability of DPSC-NPCs and SHED-NPCs after transplantation into the inner ear of rats that were treated with 10 mM ouabain to induce SGN degeneration. SGN differentiation was assessed by analyzing the number of SGNs and specific SGN protein expression. The neuronal activity of regenerated SGNs was evaluated by measuring SV2A protein expression.

## Materials and Methods

### Cell Culture

All experiments were approved by the Ethics Committee on Human Rights Related to Human Experimentation of the Faculty of Dentistry/Faculty of Pharmacy (DT/PY-IRB 2014/044.2710). We isolated, cultured, and characterized DPSCs and SHED according to our previous study. 12 13 14 The cells were cultured in growth medium with high-glucose Dulbecco's Modified Eagle Medium (DMEM; Hyclone, Logan, Utah, United States) containing 10% fetal bovine serum (Biochrom Gmbh, Berlin, Germany) and 1% penicillin/streptomycin (Gibco, Rockville, Maryland, United States) in a humidified atmosphere at 37°C containing 5% CO _2_ . Once the cells reached 80 to 90% confluence, they were subcultured and harvested using 0.05% Trypsin/ethylenediamine tetraacetic acid (Gibco).

### Neural Progenitor Cell Assays

DPSCs and SHED at passage 6 were induced to NPC by culturing in neural induction medium consisting of DMEM/F12 (Gibco), 20 ng/ml basic fibroblast growth factor (bFGF; Gibco), 20 ng/ml epidermal growth factor (EGF; Gibco), 2% B-27 (Gibco), and 1% penicillin/streptomycin.. We then plated the cells on a low-attachment culture dish (Nunclon Sphera, Thermo Fisher Scientific, Loughborough, UK) at 1.25 × 10 ^5^ cells/mL and incubated for 5 days to monitor the formation free-floating cell clusters or NPCs. The NPCs were dissociated with 1 mL of 1× Accutase enzyme (Gibco) and were incubated for 10 minutes at room temperature.

### Animal Model Creation

Ethical permission was obtained from the Faculty of Science, Animal Care, and Use Committee SC-ACUC (protocol no. SC58-011-326), and all experiments were conducted according to these guidelines. Eight-week-old male Sprague–Dawley rats with a mean weight of 250 ± 10 g were included in these experiments. The animals were housed in cages with a 12-h light-dark cycle and a temperature of 22 ± 1°C and had access to a solid diet and reverse osmosis water.

**Fig. 1:**
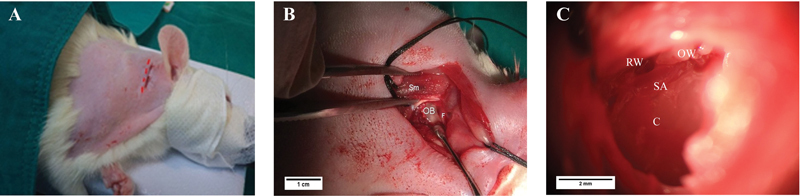
Establishing a rat hearing loss model. ( **A** ) Retroauricular incision of the right ear. ( **B** ) Otic bulla (OB), facial nerve (F), and sternocleidomastoid muscle (Sm). Scale bars: 1 cm. ( **C** ) Otic bulla area; cochlear (C), oval window (OW), round window (RW), and stapedial artery (SA). Scale bars: 2 mm.

The rats were anesthetized with an intramuscular injection of 5 mg/kg xylazine (X-lazine; L.B.S. Laboratory Ltd., Bangkok, Thailand) and 30 mg/kg tiletamine-zolazepam (zoletil100; Virbac Laboratories, Carros, France). Intraoperative anesthesia was maintained through 1 to 2% isoflurane (Sigma-Aldrich Corp., St. Louis, Missouri, United States) using a rat mask. Bullectomy and cochleostomy were performed via a retroauricular approach on the right ear ( Fig. 1A ). Following skin and subcutaneous tissue separation, the sternocleidomastoid muscle, facial nerve, and otic bulla were observed ( Fig. 1B ). The otic bulla was then drilled with a 0.5-cm diameter. We observed that the round window was located above the stapedial artery in the basal turn of the cochlea ( Fig. 1C ). The rats were injected with 10 mM ouabain octahydrate (Sigma-Aldrich) for the HL group or with sterile normal saline solution for the sham group at the round window niche. After surgery, the animals were rested on electric blankets to stay warm until they recovered. The animals received 2 to 4 mg/kg administration of carprofen per day for 3 days.

### Stem Cell Transplantation

Seven days after HL induction, the animals were anesthetized. The sham group was injected with 10 µL DMEM medium in the round window area. The HL induction groups were injected with 1 × 10 ^5^ DPSC-NPCs (HL-DPSC) or SHED-NPCs (HL-SHED) in 10 µL DMEM medium, whereas the control group was injected with 10 µL DMEM medium (HL-medium). The animals were administered cyclosporine subcutaneously (15 mg/kg; Novartis Ltd., Bangkok, Thailand) before the stem cell transplantation and each day after transplantation until the end of the experiments.

### Histological Studies

Rats were euthanized 4, 7, and 10 weeks after transplantation with 150 mg/kg thiopental (Anesthal; Jagsonpal Pharmaceuticals, New Delhi, India). The cochleae were dissected, fixed in 10% formalin, and decalcified using Decalcifier II solution (Leica Biosystems Inc., Buffalo Grove, Illinois, United States). We subsequently divided the cochleae into halves along a standardized midmodiolar plane, and the basal turn was identified by using the stapedial artery as a landmark. Tissue dehydration was performed in a gradient ethanol solution before embedding the tissues in paraffin wax. We deparaffined and rehydrated the 5-µm-thick tissue sections; the sections were stained with hematoxylin and eosin, and examined under a light microscope. The SGN density was calculated as the number of SGNs per 10,000 µm ^2^ using ImageJ software.

## Immunohistochemistry

### Neuron *-* Specific Protein Expression

The 5-µm-thick tissue sections were placed on positively charged glass slides and were heated to 60°C for 45 minutes, deparaffined, and rehydrated. We then incubated the sections with 10 mM citrate buffer (pH 6.0; Sigma) in a microwave for 5 minutes for antigen retrieval. Subsequently, the tissue sections were treated with 0.1% Triton X-100 (Sigma) at room temperature for 10 minutes to enhance the ability of antibodies to enter the cell membrane. Blocking of the nonspecific binding was performed using 2% bovine serum albumin and 5% goat serum at 4°C for 30 minutes. Each section was incubated with anti-GATA3 (BioLegend, San Diego, California, United States; 1:500 dilution) and anti-SV2A (Santa Cruz Biotechnology, Inc., Dallas, Texas, United States; 1:500 dilution) at 4°C overnight. We subsequently incubated the sections with secondary antibodies (donkey anti-goat FITC (Santa Cruz Biotechnology; 1:100 dilution) and donkey anti-goat Alexa Flour 594 (BioLegend; 1:500 dilution)) at room temperature for 60 minutes. Nuclei counterstaining was performed using 20 μg/mL 4',6-diamidino-2-phenylindole (Sigma-Aldrich) for 10 minutes.

### Antihuman Mitochondrial Expression

Endogenous peroxidase in the tissue sections was blocked for 30 minutes with 3% hydrogen peroxide in methanol. The nonspecific binding sites were blocked with 1% bovine serum albumin. We incubated each section with antihuman mitochondria (Sigma-Aldrich; 1:500 dilution) at 4°C overnight. A secondary antibody conjugate (Envision, Dako, Denmark) for 45 minutes at room temperature was incubated with the sections. The reactions were visualized by incubating the tissue sections with a 3,3′-diaminobenzidine (Sigma-Aldrich) solution containing hydrogen peroxide. Counterstaining for each section was performed using the Mayer's hematoxylin before dehydrating and mounting the sections.

### Statistical Analysis

MS Excel and SPSS v.15.0 (SPSS Inc. Chicago, Illinois, United States) were used to perform all statistical analyses. Intergroup level of significance was determined via a one-way analysis of variance and Duncan's multiple range test with 95% confidence intervals.

## Results

### Generation of Human Dental Pulp Stem Cell-Derived NPCs

**Fig. 2:**
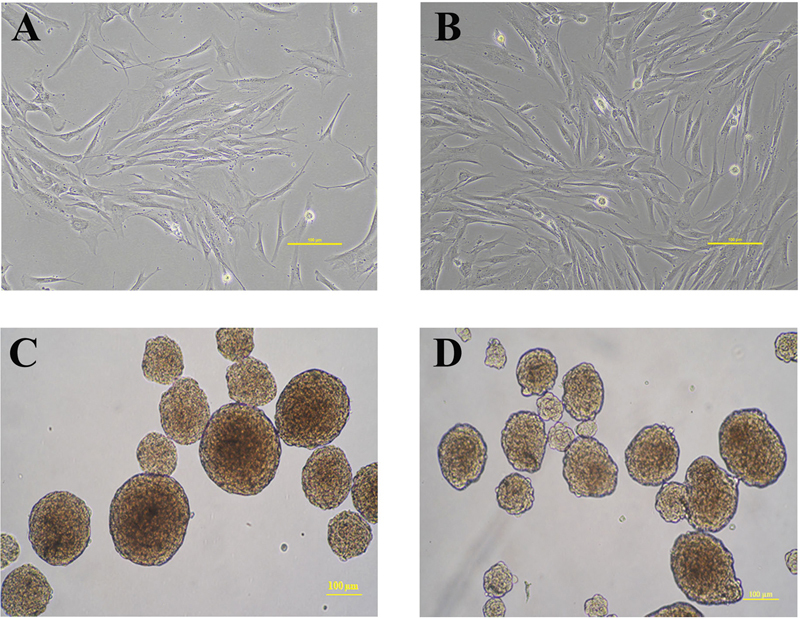
Light microscopic images showing the morphology of dental pulp stem cells (DPSCs) and neurospheres. Morphology of stem cells isolated from ( **A** ) permanent (DPSC) and ( **B** ) deciduous (SHED) teeth. Scale bars: 100 µm. Neurospheres were generated from ( **C** ) DPSCs and ( **D** ) SHED after culturing in low attachment plates for 5 days. Scale bars: 100 µm.

To induce DPSCs and SHED into neural lineages, the cells were cultured with bFGF, EGF, and B-27 on low-attachment culture dishes. Both DPSCs ( Fig. 2A ) and SHED ( Fig. 2B ) formed free-floating clusters of cells known as DPSC-NPCs ( Fig. 2C ) and SHED-NPCs ( Fig. 2D ), respectively. After induction for 5 days, NPCs with 100 to 200 µm diameter were dissociated into single cells for transplantation.

### Effect of Human DPSC Injection on SGN Regeneration

**Fig. 3:**
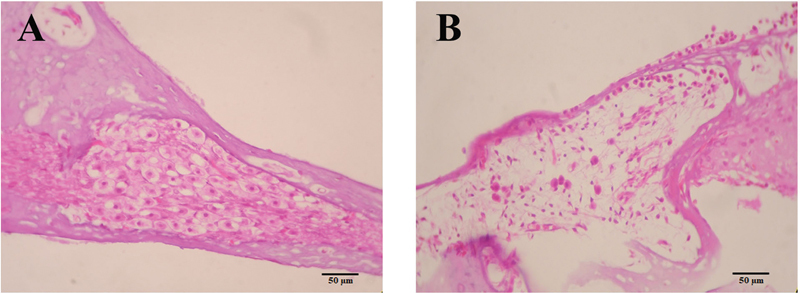
Spiral ganglion neuron morphology. ( **A** ) Hematoxylin and eosin (H&E) staining of the sham group 7 days after surgery (scale bar, 50 μm). ( **B** ) H&E staining of tissues of rats injected with 10 mM ouabain for 7 days (scale bar, 50 μm).

The purpose of the present study was to evaluate the efficacy of SGN regeneration after DPSC and SHED transplantation in a rat HL model. A total of 36 rats survived to the end of the study. After 7 days of ouabain injection, rat SGNs at the inner ear bone were severely damaged more than 85% ( Fig. 3B ) compared with the sham group ( Fig. 3A ).

**Fig. 4:**
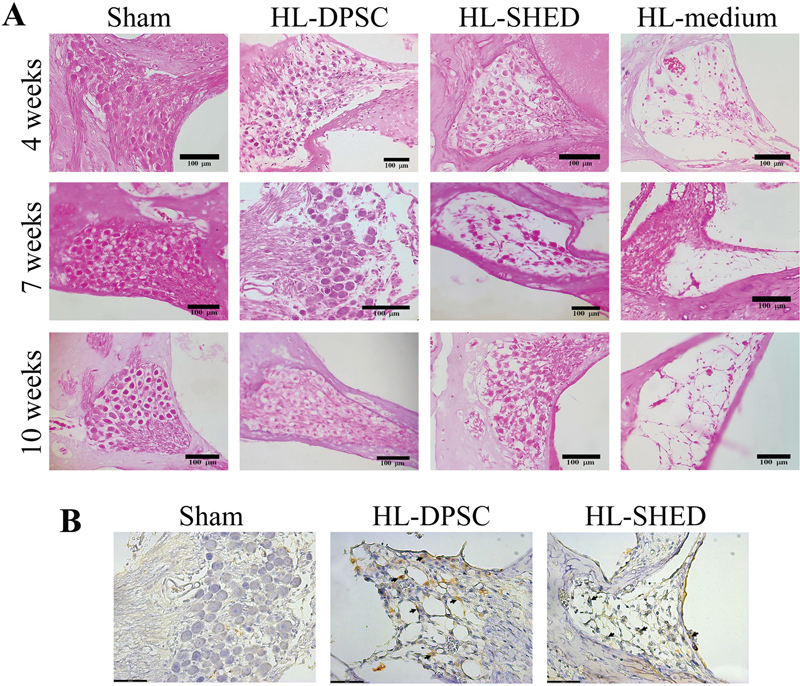
Histologic findings of spiral ganglion neurons in hearing loss rats after transplantation with DPSCs. ( **A** ) Hematoxylin and eosin staining of the spiral ganglion in rats at 4, 7, and 10 weeks after transplantation (scale bars: 100 µm). ( **B** ) Human mitochondria staining of the sham, HL-DPSC, and HL-SHED (scale bars: 100 µm) surgical areas at 10 weeks after transplantation. HL, hearing loss; DPSC, dental pulp stem cells; SHED, stem cells from exfoliated deciduous teeth.

We observed that SGNs densely packed the basal turn of the cochlea in the sham group ( Fig. 4A ), and that the mean SGN densities at 4, 7, and 10 weeks after transplantation were 22.78 ± 5.07, 20.27 ± 2.68, and 23.46 ± 2.67, respectively ( Table 1 ). In contrast, degenerating SGNs were still present in the HL-medium rats at all times ( Fig. 4A ), and the mean SGN densities at 4, 7, and 10 weeks after transplantation were 1.94 ± 0.11, 1.52 ± 0.08, and 2.29 ± 0.23, respectively ( Table 1 ). As expected, SGN regeneration was observed in the rat HL model after transplantation with both DPSCs and SHED ( Fig. 4A ). The mean SGN densities in the HL-DPSC rats at 4, 7, and 10 weeks after transplantation were 14.01 ± 0.95, 10.32 ± 1.63, and 11.04 ± 3.01, respectively ( Table 1 ). For the HL-SHED group, the mean SGN densities at 4, 7, and 10 weeks after transplantation were 14.61 ± 1.62, 11.82 ± 2.11, and 12.50 ± 2.01, respectively. The regenerated SGN density after transplantation of DPSCs and SHED was not significantly different at any time point.

**Table 1:** The mean SGN densities at 4, 7, and 10 weeks after dental pulp stem cell transplantation

Group	Mean SGN/10,000 µm ^2^ ± SD		
	4 weeks	7 weeks	10 weeks
Sham	22.78 ± 5.07 ^a^	20.27 ± 2.68 ^a^	23.46 ± 2.67 ^a^
HL-DPSC	14.01 ± 0.95 ^b^	10.32 ± 1.63 ^b^	11.04 ± 3.01 ^b^
HL-SHED	14.61 ± 1.62 ^b^	11.82 ± 2.11 ^b^	12.50 ± 2.01 ^b^
HL-medium	1.94 ± 0.11 ^c^	1.52 ± 0.08 ^c^	2.29 ± 0.23 ^c^

Abbreviations: HL, hearing loss; DPSC, dental pulp stem cells; SHED, stem cells from exfoliated deciduous teeth; SGN, spiral ganglion neuron; SD, standard deviation.

Different superscripts mean statistically significant difference ( *p* *<* 0.05).

We performed antihuman mitochondrial immunohistochemistry to confirm SGN regeneration in rats transplanted with human stem cells. The sham and HL-medium groups showed no expression of antihuman mitochondria; however, the SGN regeneration area with DPSCs and SHED showed positive immunoreactivity for human mitochondria ( Fig. 4B ).

### Expression of Neuron-Specific Proteins

**Fig. 5:**
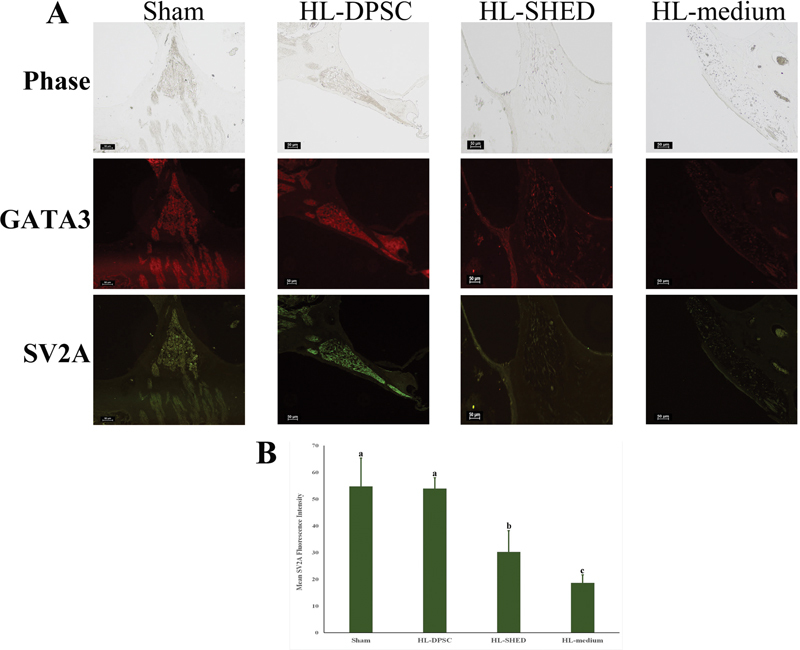
Immunofluorescence images of the spiral ganglion in hearing loss rats after transplantation with dental pulp stem cells. ( **A** ) Phase contrast of spiral ganglion (SGN) morphology, showing the protein expression of SGN markers GATA3 and SV2A in the sham, HL-DPSC, HL-SHED, and HL-medium specimens (scale bars: 50 µm) at 10 weeks after transplantation. ( **B** ) The average mean fluorescence intensity of SV2A for the spiral ganglion in the sham, HL-DPSC, HL-SHED, and HL-medium groups. HL, hearing loss; DPSC, dental pulp stem cells; SHED, stem cells from exfoliated deciduous teeth; SV2A, synaptic vesicle protein.

To identify SGNs, GATA3, a transcription factor initially expressed during the early development of SGNs, was used. Ten weeks after transplantation, the SGNs in the sham group expressed GATA3 protein, which was also detected in the regenerated cells in the HL-DPSC and HL-SHED rats (Fig. 5A). However, the fluorescence intensity of GATA3 was greater in HL-DPSC than that in HL-SHED. The GATA3 protein was not readily detected in the HL-medium group.

We aimed to determine the functional ability of SGN cells by measuring SV2A, a protein that is considered to be involved in vesicle function and neurotransmitter release, was measured to determine the. The regenerated cells at the basal turn of the cochlea in the HL-DPSC group showed similar levels of average mean SV2A fluorescence intensity in the cells of the sham group ( Fig. 5A and 5B ). Moreover, the SV2A protein in HL-DPSC exhibited significantly higher expression than the regenerated cells in the HL-medium group ( *p* < 0.05). Cells in the DPSC transplantation group exhibited significantly higher average mean SV2A fluorescence intensity than the rats that received a SHED transplant ( *p* < 0.05). Furthermore, SV2A protein expression was higher in the HL-SHED group than that in the HL-medium group ( Fig. 5A and 5B ).

## Discussion

HL is primarily caused by damage to SGNs and hair cells in the ear. 2 SGNs are a group of neuron cell bodies located in the modiolus, which is the conical central axis of the cochlea. SGN axons are projected toward the ventral and dorsal cochlear nuclei as the cochlear nerve, a vestibulocochlear nerve branch. SGNs cannot self-regenerate after degeneration and are permanently lost. 3 Stem cells can be considered suitable for cell replacement therapy and tissue repair owing to the fact that they are capable of dividing and differentiating into specialized cell types. 15 Recently, DPSCs and SHED have attracted attention in the field of regenerative medicine, particularly regarding neurodegenerative diseases. 16 Moreover, DPSCs have the potential to treat HL caused by SGN damage. DPSCs and SHED are highly effective at SGN proliferation and differentiation *in vitro* . 12 13 In this study, we demonstrated the successful regeneration of SGNs in a rat HL model using DPSCs. In a previous study, mouse embryonic stem cells were delivered to the deafened guinea pig cochlea. The transplanted cells survived 4 weeks and retained the expression of the early neuronal marker neurofilament. 17 Furthermore, human embryonic stem cells (HESs) restored the auditory neuron function in adult gerbils. 3

Positive immunoreactivity for human mitochondria in SGNs revealed that DPSCs and SHED could differentiate after following the neurogenic lineage in rat tissues. DPSCs promote the regeneration of neuron cells after implantation in Alzheimer's and Parkinson's disease models. 18 19 20 21 22 23 Mesenchymal stem cells exhibit proliferative potential in murine tissue, modulating the host immune response. Secretion of proinflammatory cytokines (interferon-gamma and tumor necrosis factor-alpha) is critical in the inflammatory cascade induced through the activation of macrophages, cytotoxic T lymphocytes, natural killer cells, and neutrophils and is reduced in stem cells. 24 In rats with HL that had receiving DPSCs and SHED transplants, we observed that the number of SGNs increased significantly at the basal turn region of the cochlea after 4 to 10 weeks of transplantation. Histopathological analysis revealed that the number of regenerated SGNs in rats transplanted with DPSCs and SHED was similar. In our previous study, we showed that DPSCs exhibited comparable plasticity toward SGNs as SHED. 12 All transplanted animals survived up to 10 weeks. Regenerated SGNs were observed from 4 to 10 weeks after transplantation in both the HL-DPSC and HL-SHED groups.

The regenerated SGNs expressed the GATA3 and SV2A proteins at 10 weeks following transplantation. According to Chen et al, histological analysis 10 weeks after HES cell transplantation in HL animals revealed that the ectopic ganglion continued to be present and appeared to express the 3A10 neurofilament-associated antigen and NKAα3, a marker of type I neurons and afferent fibers, in the inner ear. 3 Interestingly, HL rats transplanted with DPSC at 10 weeks had a statistically insignificant increase in GATA3 protein expression in SGNs than those transplanted with SHED. Moreover, a hallmark of functional ability in neurons is the expression of the synaptic vesicle glycoprotein SV2A, a protein that is involved in vesicle function and neurotransmitter release. Furthermore, we observed that HL-DPSC animals were more likely to exhibit increased SV2A fluorescence intensity compared with the HL-SHED group. Both DPSCs and SHED are capable of differentiating into functionally active neuronal cells, which have voltage-dependent sodium channels and produce an electrical potential. 23 25 26 Additionally, they also demonstrate protective effects in models of spinal cord injury, Alzheimer's disease, and retinal injury by releasing neurotrophic factors in both *in vitro* and *in vivo* experiments. 18 19 20 However, a comparison of the profiles of cytokines secreted from DPSCs revealed that the expression of some that are involved in neurogenesis, including β-nerve growth factor, betacellulin, bFGF, and NT-4, was higher in DPSCs than in SHED. 27 This may be the reason that DPSCs exhibited higher protein levels of GATA3 and SV2A than SHED. Furthermore, SHED are derived from deciduous teeth. SHED improved the recovery of motor deficits in a rat model of Parkinson's disease as early as 4 and 16 weeks after transplantation. The 4-week time point showed functional improvement in turnover, but this was not as significant as at other time points. 23 SHED may require a longer time to convert to mature SGN cells in rat HL models.

## Conclusions

This study was the first to demonstrate that both DPSCs and SHED can regenerate SGN cells in rats with SNHL. DPSCs tended to show greater SGN regeneration compared with SHED in rats with HL. These data suggest the potential of using DPSCs as a cell-based therapy for neurosensory loss in patients.
